# Inflammation Course and Skeletal Muscle Wasting Correlation During the First Week in ICU: A New Approach for Personalised Feeding of Critically Ill Patients?

**DOI:** 10.3390/jpm16080409

**Published:** 2026-07-30

**Authors:** Gilberto Gremese, Matteo Comuzzi, Matteo Danielis, Tommaso Piani, Daniele Guerino Biasucci, Giuseppe Cuttone, Ciro Fittipaldi, Francesca Lucchese, Luigi Vetrugno, Cristian Deana

**Affiliations:** 1 Anaesthesia and Intensive Care Unit 1, Department of Anaesthesia and Intensive Care, Academic Hospital of Udine, Health Integrated Agency of Friuli Centrale, 33100 Udine, Italy; matteo.comuzzi@asufc.sanita.fvg.it (M.C.); francesca.lucchese@asufc.sanita.fvg.it (F.L.); cristian.deana@asufc.sanita.fvg.it (C.D.); 2 Laboratory of Studies and Evidence Based Nursing, Department of Cardiac, Thoracic, Vascular Sciences and Public Health, University of Padua, 35128 Padua, Italy; matteo.danielis@unipd.it; 3 Health Professions Staff, Health Integrated Agency of Friuli Centrale, 33100 Udine, Italy; tommaso.piani@asufc.sanita.fvg.it; 4 Department of Clinical Science and Translational Medicine, Tor Vergata University, 00133 Rome, Italy; danielebiasucci@gmail.com; 5 Anaesthesia and Intensive Care Unit, “Abele Ajello” Hospital, ASP Trapani, 91026 Mazara del Vallo, Italy; giuseppe.cuttone@hotmail.it; 6 Anaesthesia and Intensive Care Unit, ASL Napoli 1 Centro—Ospedale del Mare, 80147 Naples, Italy; ciro.fittipaldi@aslnapoli1centro.it; 7 Anaesthesia and Intensive Care Unit, Department of Emergency, Tolmezzo Hospital, Health Integrated Agency of Friuli Centrale, 33028 Tolmezzo, Italy; luigi.vetrugno@asufc.sanita.fvg.it

**Keywords:** skeletal muscle wasting, C-reactive protein, bioelectrical impedance analysis, ICU-acquired weakness, personalised nutrition, phase angle, inflammation, muscle catabolism

## Abstract

**Background**: Critically ill patients undergo rapid and clinically significant skeletal muscle loss during the first week of ICU admission, driven by a complex interplay of systemic inflammation, neuroendocrine dysregulation, and accelerated protein catabolism. While CRP is an established marker of the inflammatory response, its temporal relationship with early muscle wasting—specifically whether the initial inflammatory peak or its subsequent persistence most strongly determines muscle loss—remains poorly characterised. This study investigated the serial dynamics of inflammatory biomarkers and their correlation with skeletal muscle changes during the first seven ICU days. **Methods**: This is a post hoc analysis of the NUTRITI prospective observational cohort, conducted at a single academic ICU in Udine, Italy (Ethics Committee approval: CEUR-2019-Os-17). Sixty-six adult critically ill patients with an anticipated ICU stay exceeding 72 h and requiring artificial nutritional support were included; patients on renal replacement therapy or with contraindications to bioelectrical impedance analysis (BIA) were excluded. Body composition—skeletal muscle mass (MM, kg) and phase angle (PA°)—was assessed by single-frequency BIA (50 kHz) on Day 1 and Day 7. The primary outcome was ΔMM%, the percentage change in muscle mass between admission and Day 7, calculated as ΔMM% = [(MMd − MMa)/MMa] × 100. Daily inflammatory biomarkers—CRP (mg/L), total WBC (×10^3^/mm^3^), and lymphocyte count (×10^3^/mm^3^)—were collected throughout. Spearman rank correlations between ΔMM% and serial biomarkers were computed for each day. Given the large number of tests (42 total), Bonferroni false discovery rate corrections were applied. **Results**: The cohort had a median age of 68.5 years (IQR 61–77.8), was predominantly male (71.2%), with median APACHE II 21.5 and SOFA 7. Median muscle mass declined significantly from 34.3 kg (IQR 29.9–39.5) at Day 1 to 30.6 kg (IQR 26.5–34.9) at Day 7 (*p* < 0.001), corresponding to a median MM% of −8.45% (IQR −14.2% to −1.52%). Phase angle also declined significantly (4.9° to 4.5°; *p* < 0.01). CRP showed no significant correlation with ΔMM% at Days 1 or 2, but a negative correlation emerged at Day 3 (ρ = −0.297; *p* = 0.020) and peaked at Day 4 (ρ = −0.355; *p* = 0.006), attenuating thereafter. CRP at Days 5 and 6 correlated with phase angle changes (*p* = 0.017 and *p* = 0.022, respectively). WBC showed no significant correlations at any time point. Day-7 lymphocyte count was nominally correlated with ΔMM% (ρ = −0.314; *p* = 0.040). Neither APACHE II nor SOFA at admission correlated with ΔMM%. No test survived correction for multiple comparisons. **Conclusions**: The kinetics of CRP—rather than its initial intensity—seem to be associated with early skeletal muscle catabolism in critically ill patients, with a temporally specific signal emerging at Days 3–4 of ICU admission. Although these findings are exploratory and did not survive multiple testing correction, their biological plausibility—grounded in the known kinetics of ubiquitin-proteasome activation and NF-κB signalling—and their alignment with the emerging concept of inflammation-guided nutritional timing both support their value as a hypothesis-generating observation. Serial CRP monitoring may represent a pragmatic candidate biomarker to identify the optimal window for nutritional escalation, pending prospective validation in adequately powered trials.

## 1. Introduction

Critical illness is invariably accompanied by profound metabolic derangements characterised by a complex neuroendocrine response and intense systemic inflammatory activation [1].

Irrespective of the underlying aetiology—whether sepsis, trauma, or acute brain injury—critically ill patients enter a state of sustained hypercatabolism and accelerated protein turnover, with skeletal muscle serving as the primary reservoir of gluconeogenic substrates for hepatic glucose production [2,3,4,5]. This catabolic response can result in clinically significant reductions in lean body mass within days [6].

Multiple interacting pathways drive this process, including proteasome-mediated proteolysis, decreased protein synthesis, and inflammation-mediated insulin resistance—all of which compound the net negative protein balance observed in these patients [7].

A systematic review and meta-analysis involving 3251 patients demonstrated that critically ill patients lose approximately 2% of skeletal muscle mass per day during the first week of ICU admission [8].

Intensive care unit-acquired weakness (ICU-AW) is a common and clinically significant neuromuscular complication of critical illness, defined as a generalised, symmetric limb and respiratory muscle weakness arising during the ICU stay, in the absence of a primary neurological cause [9].

Its pathophysiology is multifactorial and involves an imbalance between protein synthesis and degradation, mitochondrial dysfunction, alterations in sarcoplasmic reticulum function, destruction of myofilament architecture, neuropathy, and impairment of muscle satellite cells. Each of these mechanisms interacts in a different way across the diverse individuals, depending on illness severity, aetiology, and treatment exposures [10].

The principal risk factors include high illness severity at admission, sepsis, multiple organ failure, prolonged inflammation and immobilisation, and hyperglycaemia, with the contribution of corticosteroids and neuromuscular blocking agents remaining incompletely characterised [11].

The clinical consequences extend well beyond the ICU: ICU-AW is independently associated with delayed weaning from mechanical ventilation, prolonged ICU and hospital length of stay, higher in-hospital costs, and significantly increased one-year mortality, with persistence and severity of weakness at ICU discharge further amplifying long-term mortality risk [12,13,14,15].

Survivors frequently experience permanent functional disability that substantially reduces quality of life and generates a considerable burden on healthcare resources. Early physical rehabilitation interventions have the potential for mitigating ICU-AW, although randomised trials show inconsistent results, reflecting the heterogeneity of patient trajectories and the absence of personalised treatment algorithms. Indeed, the highly variable clinical phenotypes of ICU-AW—ranging from transient weakness to irreversible neuromyopathy—underscore the need for individualised monitoring strategies that integrate inflammatory, nutritional, and functional parameters to identify the higher-risk patients and to guide the timing of targeted interventions [16].

Inflammation triggers a neuroendocrine stress response characterised by adrenergic stimulation, elevated cortisol, blunted somatotropic axis activity, and hypogonadism; together, these mediators drive excessive metabolic rates, which result in protein hypercatabolism in muscle and bone, insulin resistance, and altered lipid metabolism—changes that are initially adaptive but become deleterious when sustained [17].

Bioelectrical impedance analysis (BIA)-derived parameters such as skeletal muscle mass and phase angle (PA), a surrogate of cell membrane integrity and body cell mass, have emerged as prognostically meaningful indicators in critically ill patients [18,19,20].

Recent evidence further demonstrates a strong linear relationship between changes in phase angle and daily creatinine excretion—a structural muscle mass marker—supporting the validity of BIA-derived parameters for monitoring dynamic muscle mass changes in the ICU [21]. These tools, endorsed by major international guidelines including the European Society for Clinical Nutrition and Metabolism (ESPEN 2023) and the Japanese Critical Care Nutrition Guideline (2024), offer an opportunity to integrate inflammatory monitoring with real-time body composition changes assessment [22,23].

However, the precise temporal relationship between the inflammatory burden and skeletal muscle depletion remains incompletely characterised during the first week of ICU stay: it is unclear whether the intensity of the initial inflammatory peak, the persistence of inflammation across the subacute phase, or both, most strongly determine the degree of early muscle loss [24].

Biomarkers and metabolomics have been proposed as tools to identify the transition between the acute catabolic phase and the later anabolic phase, yet no validated metabolic monitor currently exists that reliably indicates any readiness for nutritional escalation at the bedside [25].

Against this background, this study investigated the serial dynamics of inflammatory markers and early skeletal muscle wasting— measured with BIA—during the first seven days of ICU admission to verify if any correlation exists.

## 2. Materials and Methods

This study is a post hoc analysis of the NUTRITI prospective observational cohort study conducted at the Intensive Care Unit of the Azienda Ospedaliera Universitaria Friuli Centrale, Udine, Italy. The primary NUTRITI protocol was approved by the Regional Ethics Committee (Comitato Etico Unico Regionale, CEUR-2019-Os-17). The primary aim of NUTRITI was to characterise the temporal evolution of body composition and nutritional parameters during early critical illness [26]; the present analysis focuses specifically on the relationship between serial inflammatory markers and skeletal muscle loss during the first seven days of ICU stay.

The NUTRITI study was carried out at the Intensive Care Department of the Academic Hospital of Udine, and it was retrospectively registered on ClinicalTrials.gov (Identifier: NCT05473546 registered on 26 July 2022). All patients were enrolled from 1 September to 30 October 2019 and from 1 August to 30 October 2021.

Adult patients (≥18 years) admitted to the ICU for acute medical or surgical critical illness were eligible if their anticipated ICU stay exceeded 72 h and if they required artificial nutritional support (enteral or parenteral). Exclusion criteria included: inability to perform a reliable BIA measurement (e.g., presence of a cardiac implantable electronic device such as a pacemaker or defibrillator; prior limb amputation); pre-existing chronic neuromuscular disease (hereditary myopathies or dystrophies); unavailability of paired BIA measurements; and acute kidney injury requiring renal replacement therapy, owing to its potential confounding effect on fluid balance and BIA accuracy.

Baseline demographic and clinical data were recorded at ICU admission, including age, sex, admission diagnosis, body mass index (BMI), the Acute Physiology and Chronic Health Evaluation II (APACHE II) score, the Sequential Organ Failure Assessment (SOFA) score, the Nutrition Risk in the Critically Ill (NUTRIC) score, and serum albumin concentration. Inflammatory biomarkers were collected daily for the first seven days of ICU admission, including C-reactive protein (CRP, mg/L), total leukocyte count (WBC, ×10^3^/mm^3^), and lymphocyte count (Ly, ×10^3^/mm^3^).

Blood exam tests (CRP, WBC, lymphocyte) measurements were performed at the central laboratory, which complies with international standard quality criteria.

Body composition was assessed by single-frequency BIA at 50 kHz using a validated device (EFG V.3; Akern, Via Lisbona 32, 50065 Pontassieve (Florence)—Italy, akern@akern.com). Measurements were performed under standardised conditions as follows:

After the patient had remained in a supine position with arms separated from trunk by about 30° and legs separated by about 45° for at least 5 min and after the skin was cleaned to ensure good contact, appropriate electrodes were attached to the right hand and foot. Enteral nutrition was stopped at least 2 h before measurement [26].

A constant alternating current of 400 µA was applied at the operating frequency of 50 kHz. The following bioelectrical parameters were directly recorded: resistance (R), reactance (Xc) and phase angle (PA). Skeletal muscle mass (MM, kg) was derived from validated internal device equations after entering anthropometric data. BIA assessments were performed on Day 1 (ICU admission) and Day 7. The change (ΔMM%) between MM at ICU admission (MMa) and at day 7 of ICU stay (MMd) is expressed as percentage according to the following ΔMM% = ((MMd − MMa)/MMa) × 100.

Single-frequency BIA at 50 kHz was preferred over multi-frequency or segmental approaches due to its validated use in the ICU setting, ease of bedside application in critically ill patients, and established reproducibility with the device used in the primary NUTRITI protocol.

Given the intrinsically non-Gaussian distribution of ICU clinical data and the presence of potential outliers, a predominantly non-parametric statistical approach was adopted. Descriptive statistics are reported as median [interquartile range (IQR)]. Paired within-patient differences in BIA parameters between Day 1 and Day 7 were assessed using the Wilcoxon signed-rank test. Bivariate correlations between ΔMM% and serial inflammatory markers or severity scores were assessed using Spearman’s rank test or Pearson’s test according to the normality of distribution assessed with the Shapiro–Wilk test. Correlations between ΔPA° were similarly tested. Bonferroni correction was applied for multiple testing.

Statistical significance was defined as *p* < 0.05. All analyses were performed using standard statistical software (Jamovi Version 2.6.44.0).

## 3. Results

### 3.1. Patient Characteristics

The analytical cohort comprised 66 adult critically ill patients. Demographic and clinical characteristics at admission are summarised in Table 1.

Admission diagnoses included respiratory failure (24.2%), neurological disease (19.7%), trauma (13.6%), cardiovascular disease (9.1%), sepsis (6.1%), post-operative monitoring (16.7%), and other causes (10.6%). Daily caloric intake (from enteral nutrition, parenteral nutrition, intravenous glucose, and propofol) and protein delivery were recorded throughout the study period as descriptive variables. Because no association between cumulative caloric or protein delivery and BIA-derived muscle changes was demonstrated in the primary NUTRITI analysis, nutritional variables were not included as covariates in the present post hoc correlation analysis.

The median age was 68.5 [61–77.8] years, and the cohort was predominantly male (71.2%). Median BMI was 27 [24.3–29] kg/m^2^. Disease severity was characterised by a median APACHE II score of 21.5 [16.8–25] and a median SOFA score of 7 [5.75–8]. Median serum albumin at admission was 29 [26,27,28,29,30,31,32] g/dL. The median ICU length of stay was 8 [5.5–17.5] days. Overall mortality at 30 days after ICU admission was 21%.

### 3.2. Body Composition Changes

Patients’ median skeletal muscle mass decreased from 34.3 [29.9–39.5] kg at day 1 to 30.6 [26.5–34.9] kg at day 7 (*p* < 0.001). The median percentage change in muscle mass (MM%) was −8.45% [−14.2% to −1.52%].

Phase angle also declined significantly between Day 1, median 4.9° [4.5–5.5], and Day 7, median 4.5° [4.0–5.0] (*p* < 0.01), with a median change of −0.25° [−1.08 to +0.1].

A strong positive correlation was observed between ΔMM% and the change in phase angle (ΔPA°) between Day 1 and Day 7 (ρ = 0.625; *p* < 0.001).

**Figure 1:**
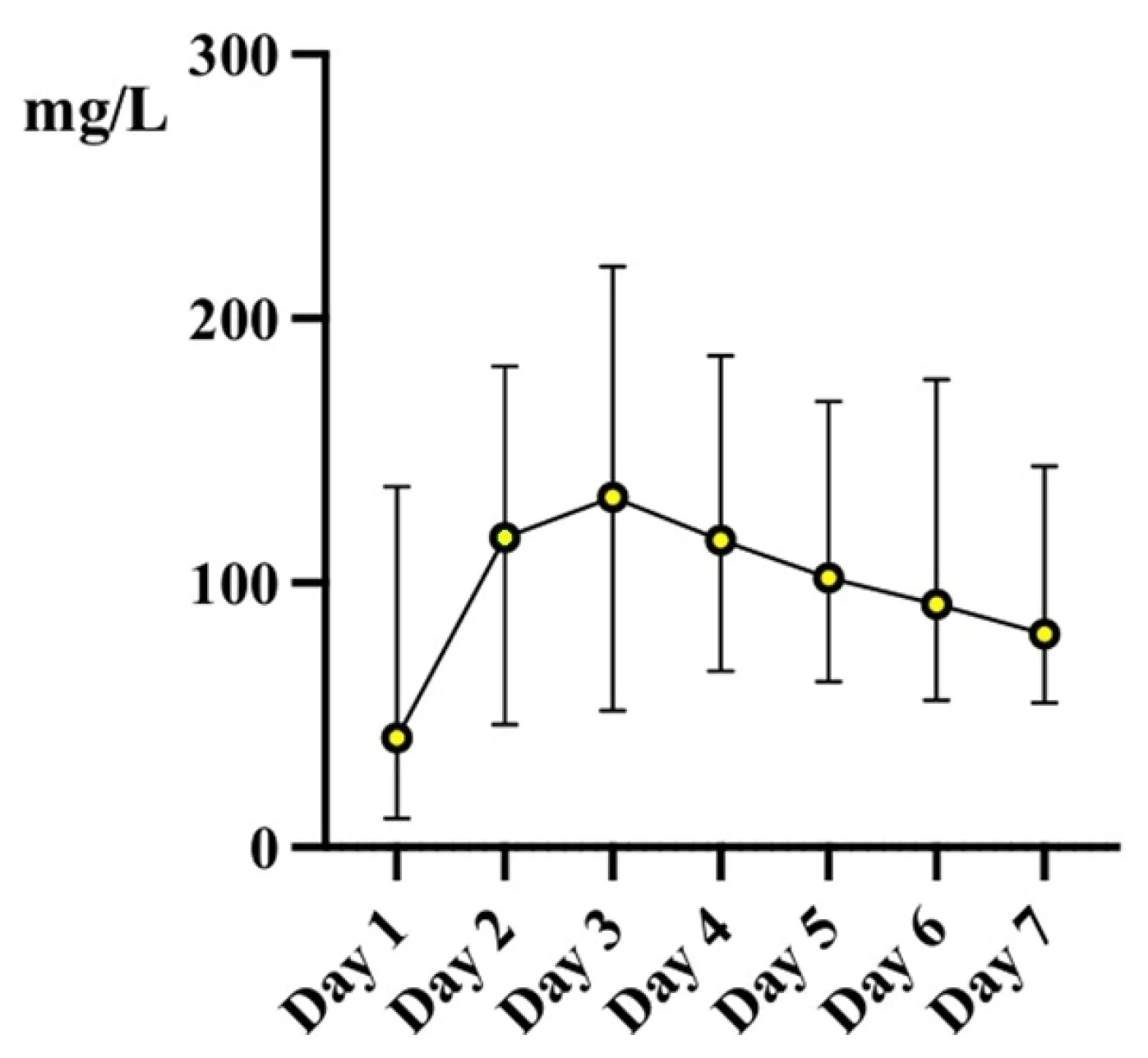
CRP levels during the first 7 days of ICU stay.

**Figure 2:**
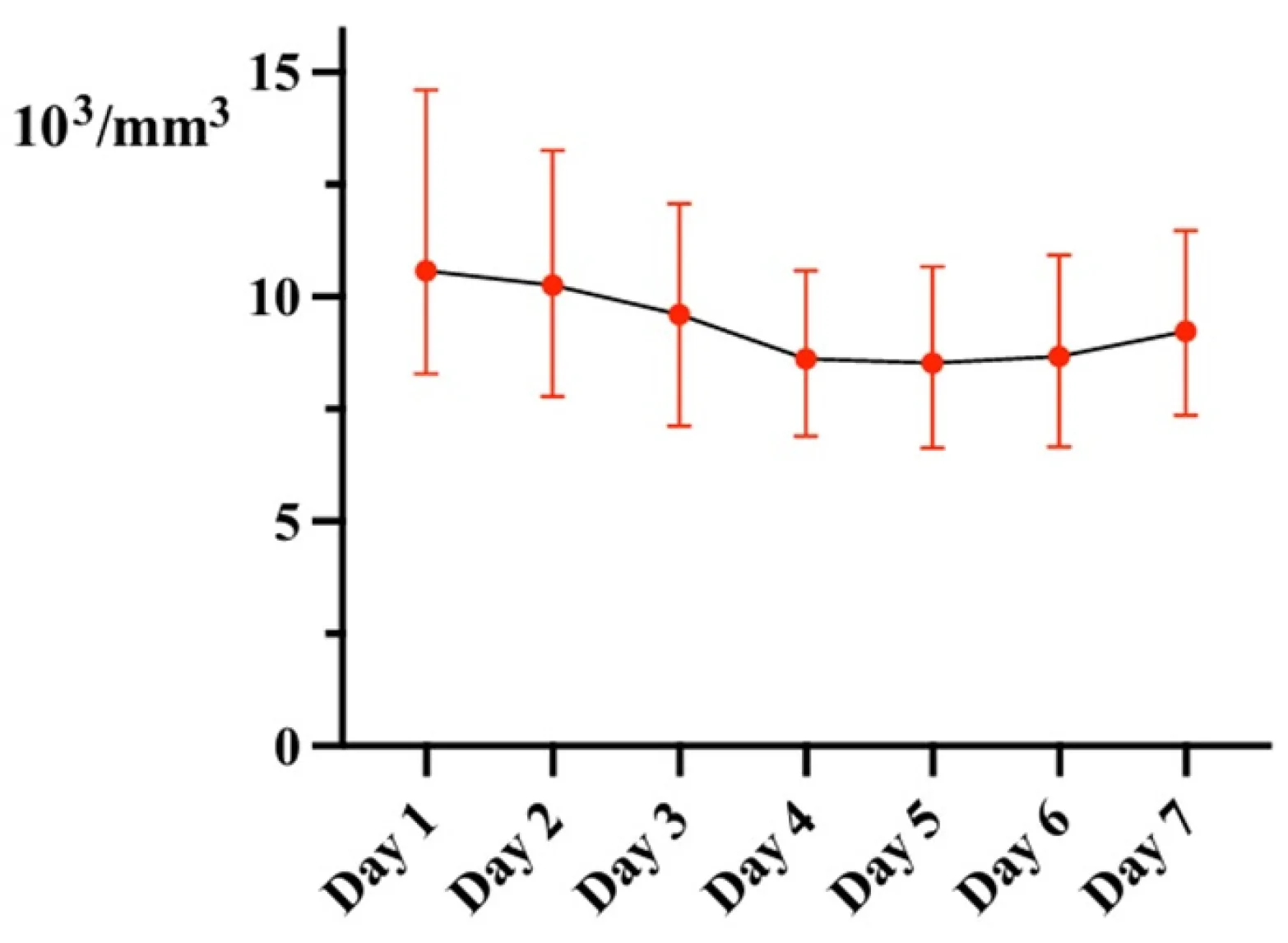
WBC levels during the first 7 days of ICU stay.

**Figure 3:**
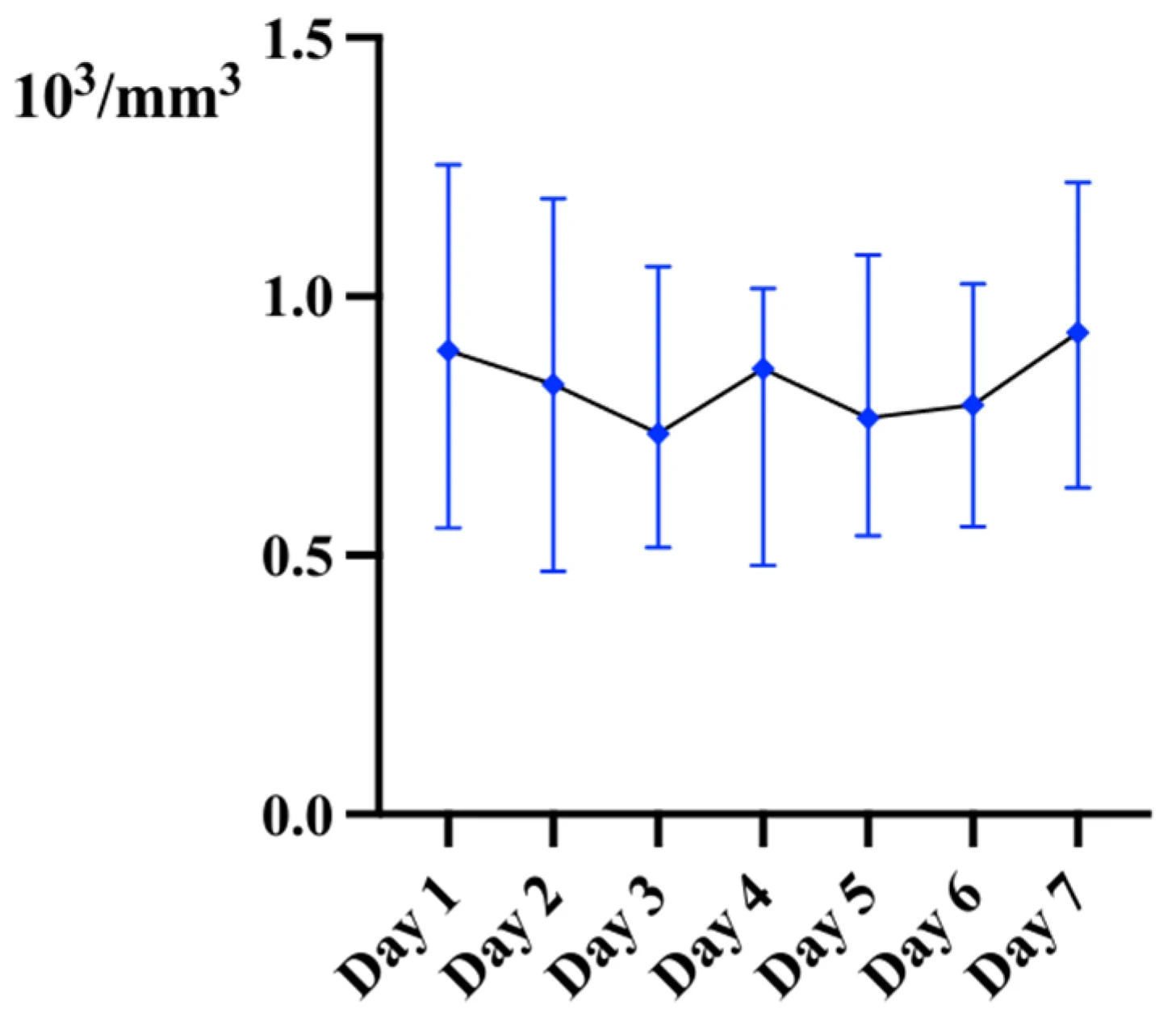
Lymphocyte count during the first 7 days of ICU stay.

Regarding inflammatory markers, CRP levels (median and IQR25–75), WBC count (median and IQR25–75), and lymphocyte count (median and IQR25–75) during the first 7 days of ICU stay are shown in Figure 1, Figure 2 and Figure 3, respectively.

### 3.3. Correlation Between Inflammatory Markers and Muscle Loss

Results of Spearman correlation analyses are summarised in Table 2.

CRP at Day 1 and 2 did not show correlation with MM%. In contrast, a statistically significant negative correlation between ΔMM% and CRP emerged at Day 3 (ρ = −0.297; *p* = 0.020) and was strongest at Day 4 (ρ = −0.355; *p* = 0.006) as shown in Supplementary Figure S1. The Day 5 correlation approached but did not reach significance (ρ = −0.269; *p* = 0.060), and at Days 6 and 7 CRP was not significantly correlated with ΔMM%.

CRP measurements were performed in the hospital central laboratory using the same standardised immunoturbidimetric assay throughout the study period.

A higher lymphocyte count at Day 7 was significantly correlated with ΔMM% (ρ = −0.314; *p* = 0.040).

Some correlations were also found between ΔPA° and CRP level on Days 5 and 6 (as shown in Table 2 and Supplementary Figure S2).

Correlations after Bonferroni correction are reported in Supplementary Table S1.

Neither APACHE II score (ρ = −0.165; *p* = 0.187) nor SOFA score (r = −0.142; *p* = 0.245) at admission were significantly correlated with the magnitude of early muscle wasting.

## 4. Discussion

This study provides prospective evidence that the temporal dynamics of inflammation—rather than its initial intensity—are correlated with early skeletal muscle wasting in critically ill patients. CRP correlation is temporally deferred, peaking at Days 3–4.

The absence of any significant association between CRP at Day 1 or Day 2 and ΔMM% suggests that the temporal dynamics of inflammation—rather than its initial intensity—may be more relevant to early muscle catabolism. A negative correlation emerged only at Day 3 (ρ = −0.297; *p* = 0.020) and reached its nadir at Day 4 (ρ = −0.355; *p* = 0.006), attenuating thereafter. We acknowledge that these associations did not survive correction for multiple comparisons and must, therefore, be interpreted as hypothesis-generating rather than confirmatory. Nevertheless, three aspects support their biological plausibility.

First, the temporal specificity is noteworthy: while prior studies have documented elevated CRP in ICU patients with greater muscle loss, none—to our knowledge—has identified a specific first-week window during which CRP kinetics most strongly track the magnitude of muscle mass loss measured by BIA [27].

The existing literature has relied predominantly on admission CRP or mean ICU values as static snapshots, without resolving day-specific temporal dynamics [28].

Second, the Days 3–4 window is biologically plausible—though not mechanistically demonstrated by this study—given that experimental evidence suggests the ubiquitin-proteasome system may require approximately 48–72 h of sustained NF-κB activation downstream of IL-6 and TNF-α to reach the full proteolytic magnitude [29,30,31], and that mTORC1 suppression by pro-inflammatory mediators [32,33,34] and myostatin induction [35] operate on a comparable timescale.

These pathways were not directly measured in the present study; this mechanistic interpretation is therefore speculative and is offered solely as a biological framework to motivate future mechanistic research.

Third, serial CRP kinetics have recently been proposed as a pragmatic surrogate for the catabolic-to-anabolic metabolic transition—a concept supported by Gargi et al. [25], who demonstrated that CRP trajectory may guide nutritional escalation decisions. Nutritional support provided during peak catabolism may be ineffective due to anabolic resistance [36] and may potentially be harmful [37].

Therefore, identifying the optimal nutritional timing via routinely available bedside biomarkers such as CRP represents a clinically actionable research question that prospective interventional trials are required to answer.

The attenuation of the correlation at Days 6–7 may reflect either the establishment of irreversible muscle loss, or the emergence of heterogeneous recovery trajectories that dilute the signal. Notably, phase angle changes correlated with CRP at Days 5–6 rather than Days 3–4, consistent with the distinct biology of these parameters: while ΔMM% captures total protein loss, phase angle reflects sarcolemmal integrity—a compartment compromised more gradually by mitochondrial dysfunction and lipid peroxidation in the subacute phase [18,38,39].

The absence of WBC correlations across all seven days confirms that non-specific leukocytosis—driven by demargination and stress granulopoiesis—is not a meaningful proxy for the catabolic inflammatory signal.

CRP, as a readout of the IL-6-governed hepatic acute-phase response [40], is a substantially more specific indicator of the cytokine milieu driving proteolytic gene transcription in muscle.

The nominal correlation between Day-7 lymphocyte counts and ΔMM% (ρ = −0.314; *p* = 0.040; *p* = 0.386 after Bonferroni correction)—indicating that higher lymphocyte counts associate with greater muscle loss—is counterintuitive and intriguing, though it must be interpreted with particular caution given the non-significance after multiple testing correction.

The mechanisms underlying this counterintuitive association remain speculative and were not investigated in the present study. Possible explanations include persistent adaptive immune activation driving pro-inflammatory cytokine release, substrate competition between lymphocytes and muscle for glutamine, or co-dependence of both variables on overall disease severity. These hypotheses require dedicated mechanistic investigation—including lymphocyte subset analysis and cytokine profiling—before any conclusions can be drawn [29,41,42,43,44,45].

Admission severity scores do not predict muscle wasting. Neither APACHE II nor SOFA correlated significantly with ΔMM%, which is mechanistically justified: these tools were designed to predict mortality and organ dysfunction [46,47], not to capture the determinants of muscle catabolism—immobility, nutritional delivery, sedation regimens, and inflammatory kinetics—none of which are reflected in point-of-care admission scores.

Considerable inter-individual variability was observed in the magnitude of muscle loss: while some patients experienced reductions exceeding 30–40% of baseline muscle mass by Day 7, a minority showed minimal change or near-stability, likely reflecting heterogeneity in baseline nutritional reserve, illness trajectory, and fluid balance dynamics. This wide distribution underscores the limitations of group-level estimates and reinforces the rationale for individualised monitoring strategies in the ICU.

Some limitations include the single-centre secondary design and modest sample size, limiting statistical power and generalisability; fluid-balance confounding on BIA accuracy, partially mitigated by concordant phase angle changes; the observational nature precluding causal inference; BIA measurement only at Days 1 and 7, preventing daily trajectory characterisation; and exclusion of patients on renal replacement therapy, introducing selection bias toward a lower-risk subgroup; corticosteroid exposure was not systematically collected and therefore could not be included in the analysis.

Multivariable adjustment for nutritional, pharmacological, and clinical confounders was not performed in the present analysis given the limited sample size and the exploratory nature of the study; such adjustment would require a substantially larger cohort and is planned in a prospective follow-up study. Finally, the large number of correlation analyses performed represents an important methodological limitation. Despite applying Bonferroni correction, the possibility that the unadjusted Days 3–4 CRP findings represent a type I error cannot be excluded. All findings must therefore be considered hypothesis-generating and require prospective confirmation in adequately powered trials.

Regarding BIA accuracy, fluid shifts, capillary leak, oedema, and dynamic changes in extracellular water—all prevalent during the first ICU week—are recognised sources of measurement bias in BIA-derived body composition estimates [6]. Although patients on renal replacement therapy were excluded and measurements were performed under standardised conditions, residual confounding from fluid status cannot be excluded and may have influenced the observed muscle mass changes. The concordant decline in phase angle, which is comparatively less sensitive to extracellular fluid accumulation, provides partial reassurance but does not fully resolve this concern.

Last, we did not have clinical follow-up regarding muscular mass and function after ICU discharge due to limited research resources. We acknowledge that this should be part of a multimodal treatment to reduce the burden of negative outcomes after ICU.

## 5. Conclusions

The Day 3–4 CRP window, if confirmed prospectively in larger, adequately powered trials, could reorient nutritional intervention design toward the subacute inflammatory phase. Leucine-enriched supplementation [48], HMB [49], and NF-κB modulation [29,50] are biologically most actionable within this timeframe. Day-7 lymphocyte count, interpreted cautiously—particularly given the absence of significance after multiple testing correction—warrants evaluation as a composite signal of inflammatory and immunological trajectory in future nutritional trials. In conclusion, serial CRP monitoring during the ICU stay—rather than admission severity scores—identifies a temporally specific window potentially associated with muscle catabolism in critically ill patients. Although these proof-of-concept findings require prospective validation before clinical translation, they support a shift toward dynamic inflammatory monitoring as the basis for a personalised nutritional strategy in critical illness, and motivate future studies powered to formally test the Days 3–4 CRP hypothesis.

## Figures and Tables

**Table 1:** Demographic and clinical characteristics at ICU admission.

Variable	Median [IQR25–75]
Patients included (N)	66
Age (years)	68.5 [61–77.8]
Male sex (n, %)	47 (71.2%)
BMI at admission (kg/m^2^)	27 [24.3–29]
APACHE II Score	21.5 [16.8–25]
SOFA Score	7 [5.75–8]
NUTRIC Score	5.5 [4–7]
Albumin at admission (g/dL)	29 [26–32]
Duration of MV (days)	6.5 [4–10]
ICU length of stay (days)	8 [6–17.5]
Cumulative fluid balance at day 7 (mL)	−1869 [−4568–+794]
CRP at admission	41.3 [20–101]

IQR = interquartile range; BMI = body mass index; APACHE = Acute Physiology and Chronic Health Evaluation; SOFA = Sequential Organ Failure Assessment; NUTRIC = Nutrition Risk in the Critically Ill; MV = mechanical ventilation.

**Table 2:** Spearman’s correlation analysis: ΔMM% vs. Serial Inflammatory Variables and Severity Scores.

	ΔMM%				ΔPA°			
Inflammation Parameter	ρ	95% CI Lower	95% CI Upper	*p*-Value	ρ	95% CI Lower	95% CI Upper	*p*-Value
CRP Day 1	−0.003	−0.248	0.243	0.984	0.13	−0.120	0.364	0.307
CRP Day 2	−0.137	−0.413	0.067	0.269	0.048	−0.202	0.292	0.708
CRP Day 3	−0.297	−0.510	−0.048	0.020 *	−0.107	−0.350	0.148	0.41
CRP Day 4	−0.355	−0.562	−0.106	0.006 **	−0.237	−0.467	0.022	0.073
CRP Day 5	−0.269	−0.520	0.008	0.060	−0.318	−0.536	−0.060	0.017 *
CRP Day 6	−0.193	−0.453	0.096	0.188	−0.330	−0.562	−0.051	0.022 *
CRP Day 7	−0.217	−0.324	0.262	0.113	−0.258	−0.513	0.038	0.087
WBC Day 1	−0.109	−0.342	0.136	0.382	0.16	−0.085	0.387	0.199
WBC Day 2	−0.208	−0.430	0.038	0.096	−0.003	−0.246	0.242	0.984
WBC Day 3	−0.078	−0.314	0.167	0.532	0.052	−0.192	0.291	0.676
WBC Day 4	−0.146	−0.380	0.106	0.255	−0.095	−0.335	0.156	0.457
WBC Day 5	−0.03	−0.284	0.227	0.819	−0.077	−0.326	0.183	0.563
WBC Day 6	0.025	−0.247	0.293	0.858	−0.047	−0.314	0.226	0.736
WBC Day 7	−0.16	−0.425	0.130	0.278	−0.233	−0.485	0.054	0.111
Ly Day 1	0.016	−0.248	0.278	0.904	0.002	−0.261	0.265	0.989
Ly Day 2	−0.02	−0.274	0.238	0.882	−0.106	−0.353	0.154	0.423
Ly Day 3	−0.085	−0.341	0.182	0.531	−0.134	−0.383	0.134	0.326
Ly Day 4	0.023	−0.233	0.275	0.863	0.139	−0.120	0.379	0.291
Ly Day 5	0.008	−0.260	0.275	0.953	0.062	−0.210	0.324	0.658
Ly Day 6	−0.103	−0.376	0.187	0.487	0.095	−0.195	0.369	0.522
Ly Day 7	−0.314	−0.562	−0.015	0.040 *	−0.283	−0.538	0.019	0.066

Legend: ΔMM% = percentage change in skeletal muscle mass; ΔPA = change in phase angle; CRP = C-reactive protein; WBC = white blood cell; Ly = lymphocyte count. * *p*  <  0.05; ** *p*  <  0.01 (unadjusted). 95% confidence intervals for all correlation coefficients are reported in Supplementary Table S1, together with Bonferroni-adjusted *p*-values. No test reached significance after correction for multiple comparisons. All findings should be considered exploratory and hypothesis-generating.

## Data Availability

The data presented in this study are available on reasonable request from the corresponding author.

## Supplementary Materials

The following supporting information can be downloaded at: https://www.mdpi.com/article/10.3390/jpm16080409/s1. Supplementary Figure S1: Scatterplots showing the negative correlation between ΔMM% and CRP and the negative correlation between lymphocyte count at Day 7 and ΔMM; Supplementary Figure S2: Scatterplots showing the negative correlation between ΔPA° and CRP level at day 5 and 6; Supplementary Table S1: Multiple comparisons after Bonferroni correction.

## Abbreviations

The following abbreviations are used in this manuscript:

APACHE II	Acute Physiology and Chronic Health Evaluation II
BIA	Bioelectrical Impedance Analysis
BMI	Body Mass Index
CRP	C-Reactive Protein
ΔMM%	Percentage change between MM at ICU admission and at Day 7 of ICU stay
ΔPA°	Delta Phase Angle (change in phase angle in degrees between Day 1 and 7)
HMB	β-Hydroxy β-Methylbutyrate
ICU	Intensive Care Unit
IFN-γ	Interferon Gamma
IL-6	Interleukin-6
IRIS	Immune Reconstitution Inflammatory Syndrome
Ly	Lymphocyte Count
MM	Muscle Mass
mTORC1	Mechanistic Target of Rapamycin Complex 1
NF-κB	Nuclear Factor kappa-light-chain-enhancer of activated B cells
NK	Natural Killer Cells
NUTRIC	Nutrition Risk in the Critically Ill
PA	Phase Angle
SOFA	Sequential Organ Failure Assessment
STAT3	Signal Transducer and Activator of Transcription 3
TNF-α	Tumour Necrosis Factor Alpha
WBC	White Blood Cell Count
