# Supplementary material for: Inflammation Course and Skeletal Muscle Wasting Correlation During the First Week in ICU: A New Approach for Personalised Feeding of Critically Ill Patients?
Source: J Pers Med. 2026 Jul 30;16(8):409. doi: 10.3390/jpm16080409 (PMC13514688; doi:10.3390/jpm16080409)
Supplement: Supplementary file 1 [file jpm-16-00409-s001.zip › SUPPLEMENTARY FIGURE S1.pdf]

Supplementary Figure S1. Scatterplots showing the negative correlation between ΔMM% and CRP, which emerged at Day 3 (ϱ= -0.297; p = 0.020) (panel A) and got stronger at Day 4 (ϱ = -0.355; p = 0.006) (panel B). Negative correlation between lymphocyte count at Day 7 and ΔMM% (ϱ=-0.314; p= 0.040) is illustrated in panel C.

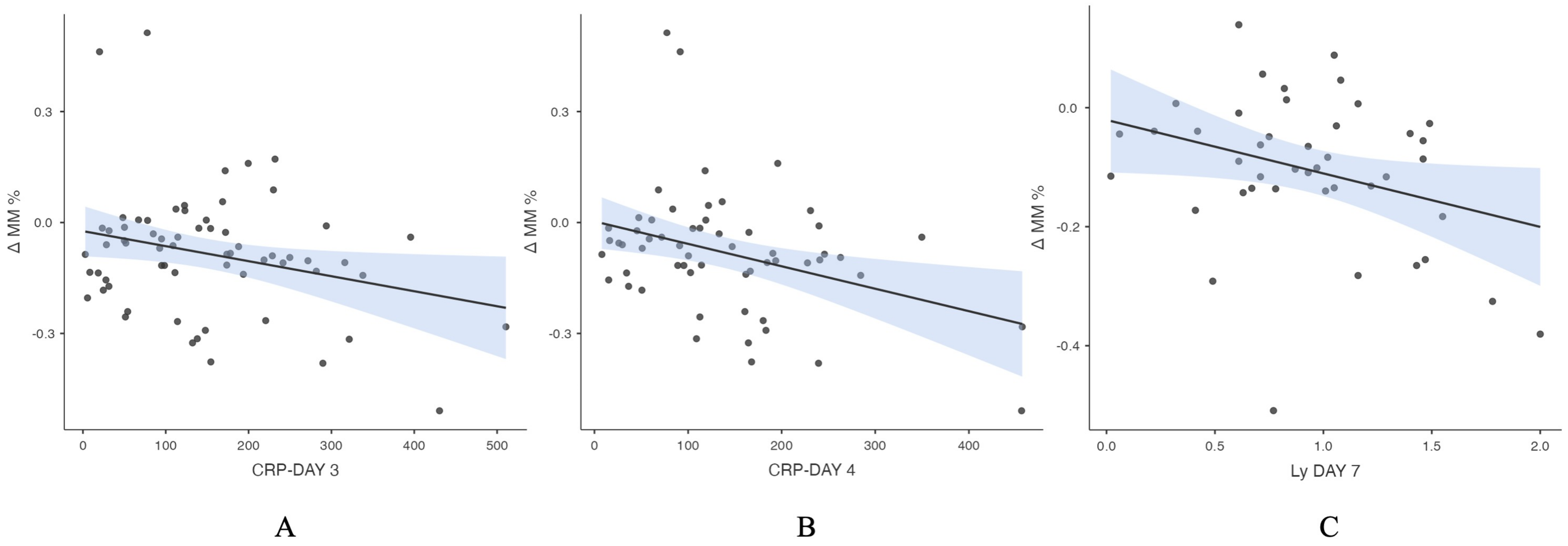

Legend: ΔMM% = percentage change in skeletal muscle mass; ΔPA = change in phase angle; CRP = C reactive protein; WBC = white blood cell; Ly = lymphocyte count.
