# Supplementary material for: Inflammation Course and Skeletal Muscle Wasting Correlation During the First Week in ICU: A New Approach for Personalised Feeding of Critically Ill Patients?
Source: J Pers Med. 2026 Jul 30;16(8):409. doi: 10.3390/jpm16080409 (PMC13514688; doi:10.3390/jpm16080409)
Supplement: Supplementary file 1 [file jpm-16-00409-s001.zip › SUPPLEMENTARY FIGURE S2.pdf]

**Supplementary Figure S2.** Scatterplots showing the negative correlation between ΔPA° and CRP level at day 5 (panel A) and 6 (panel B).

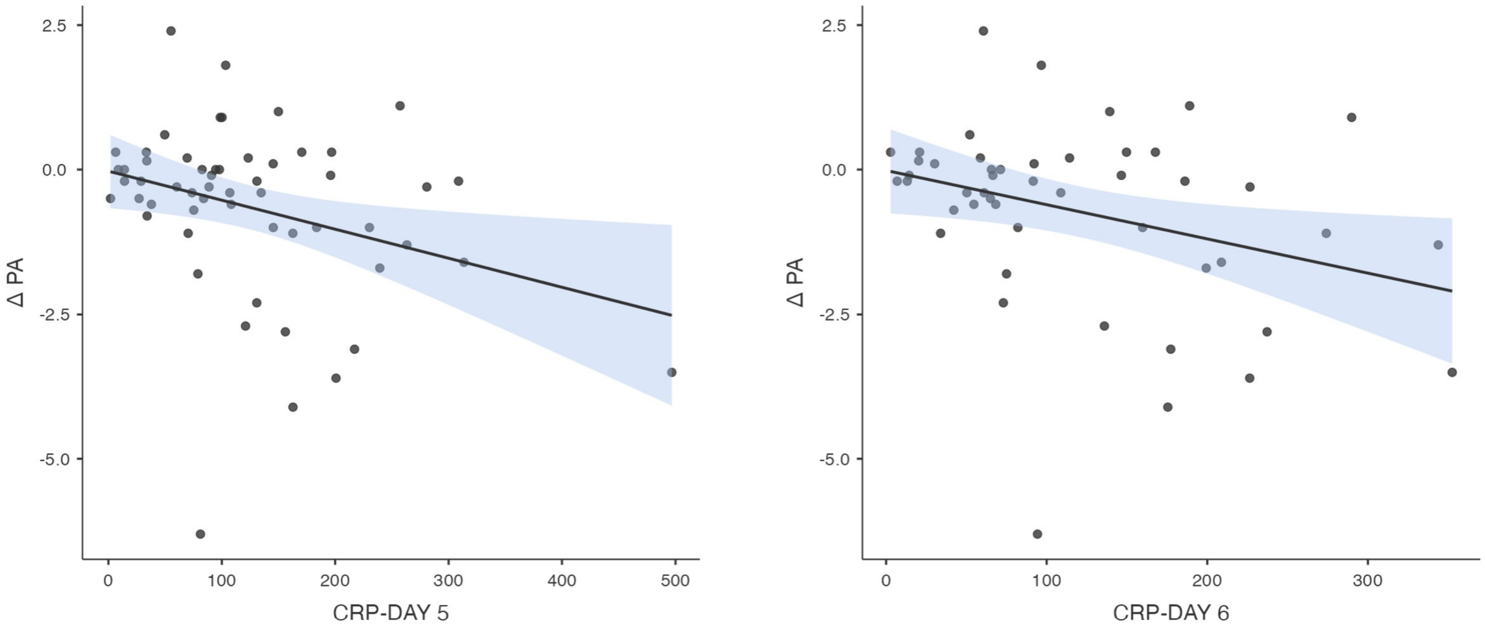


A

B

Legend: ΔPA = change in phase angle; CRP = C reactive protein.
