# Supplementary material for: Inflammation Course and Skeletal Muscle Wasting Correlation During the First Week in ICU: A New Approach for Personalised Feeding of Critically Ill Patients?
Source: J Pers Med. 2026 Jul 30;16(8):409. doi: 10.3390/jpm16080409 (PMC13514688; doi:10.3390/jpm16080409)
Supplement: Supplementary file 1 [file jpm-16-00409-s001.zip › SUPPLEMENTARY TABLE S1.pdf]

Supplementary Table S1. Multiple comparisons after Bonferroni correction.

	ΔMM%					ΔPA°				
**Inflammation parameter**	**ϱ**	**95% CI lower**	**95% CI upper**	**p-value**	**p (Bonferroni)**	**ϱ**	**95% CI lower**	**95% CI upper**	**p-value**	**p (Bonferroni)**
**CRP Day 1**	-0.003	-0.248	0.243	0.984	0.9889	0.13	-0.120	0.364	0.307	0.7168
**CRP Day 2**	-0.137	-0.413	0.067	0.269	0.5194	0.048	-0.202	0.292	0.708	0.9590
**CRP Day 3**	-0.297	-0.510	-0.048	**0.020***	0.1150	-0.107	-0.350	0.148	0.41	0.8066
**CRP Day 4**	-0.355	-0.562	-0.106	**0.006****	0.0780	-0.237	-0.467	0.022	0.073	0.3827
**CRP Day 5**	-0.269	-0.520	0.008	0.060	0.2150	-0.318	-0.536	-0.060	**0.017***	0.2150
**CRP Day 6**	-0.193	-0.453	0.096	0.188	0.5976	-0.330	-0.562	-0.051	**0.022***	0.2150
**CRP Day 7**	-0.217	-0.324	0.262	0.113	0.9889	-0.258	-0.513	0.038	0.087	0.4049
**WBC Day 1**	-0.109	-0.342	0.136	0.382	0.8024	0.16	-0.085	0.387	0.199	0.5976
**WBC Day 2**	-0.208	-0.430	0.038	0.096	0.4049	-0.003	-0.246	0.242	0.984	0.9889
**WBC Day 3**	-0.078	-0.314	0.167	0.532	0.8272	0.052	-0.192	0.291	0.676	0.9471
**WBC Day 4**	-0.146	-0.380	0.106	0.255	0.7134	-0.095	-0.335	0.156	0.457	0.8272
**WBC Day 5**	-0.03	-0.284	0.227	0.819	0.9889	-0.077	-0.326	0.183	0.563	0.8448
**WBC Day 6**	0.025	-0.247	0.293	0.858	0.9889	-0.047	-0.314	0.226	0.736	0.9664
**WBC Day 7**	-0.16	-0.425	0.130	0.278	0.7168	-0.233	-0.485	0.054	0.111	0.4223
**Ly Day 1**	0.016	-0.248	0.278	0.904	0.9889	0.002	-0.261	0.265	0.989	0.9889
**Ly Day 2**	-0.02	-0.274	0.238	0.882	0.9889	-0.106	-0.353	0.154	0.423	0.8066
**Ly Day 3**	-0.085	-0.341	0.182	0.531	0.8272	-0.134	-0.383	0.134	0.326	0.7206
**Ly Day 4**	0.023	-0.233	0.275	0.863	0.9889	0.139	-0.120	0.379	0.291	0.7168
**Ly Day 5**	0.008	-0.260	0.275	0.953	0.9889	0.062	-0.210	0.324	0.658	0.9471
**Ly Day 6**	-0.103	-0.376	0.187	0.487	0.8272	0.095	-0.195	0.369	0.522	0.8272
**Ly Day 7**	-0.314	-0.562	-0.015	**0.040***	0.2814	-0.283	-0.538	0.019	0.066	0.3827

Legend: **ΔMM%** = percentage change in skeletal muscle mass; ΔPA = change in phase angle; CRP = C reactive protein; WBC = white blood cell; Ly = lymphocyte count, CI = confidence interval.
